# The toxic metal hypothesis for neurological disorders

**DOI:** 10.3389/fneur.2023.1173779

**Published:** 2023-06-23

**Authors:** Roger Pamphlett, David P. Bishop

**Affiliations:** ^1^Department of Pathology, Brain and Mind Centre, School of Medical Sciences, The University of Sydney, Sydney, NSW, Australia; ^2^Department of Neuropathology, Royal Prince Alfred Hospital, Camperdown, NSW, Australia; ^3^Hyphenated Mass Spectrometry Laboratory, School of Mathematical and Physical Sciences, University of Technology Sydney, Sydney, NSW, Australia

**Keywords:** toxic metals, astrocytes, oligodendrocytes, locus ceruleus, multiple sclerosis, amyotrophic lateral sclerosis, Parkinson disease, Alzheimer disease

## Abstract

Multiple sclerosis and the major sporadic neurogenerative disorders, amyotrophic lateral sclerosis, Parkinson disease, and Alzheimer disease are considered to have both genetic and environmental components. Advances have been made in finding genetic predispositions to these disorders, but it has been difficult to pin down environmental agents that trigger them. Environmental toxic metals have been implicated in neurological disorders, since human exposure to toxic metals is common from anthropogenic and natural sources, and toxic metals have damaging properties that are suspected to underlie many of these disorders. Questions remain, however, as to how toxic metals enter the nervous system, if one or combinations of metals are sufficient to precipitate disease, and how toxic metal exposure results in different patterns of neuronal and white matter loss. The hypothesis presented here is that damage to selective locus ceruleus neurons from toxic metals causes dysfunction of the blood–brain barrier. This allows circulating toxicants to enter astrocytes, from where they are transferred to, and damage, oligodendrocytes, and neurons. The type of neurological disorder that arises depends on (i) which locus ceruleus neurons are damaged, (ii) genetic variants that give rise to susceptibility to toxic metal uptake, cytotoxicity, or clearance, (iii) the age, frequency, and duration of toxicant exposure, and (iv) the uptake of various mixtures of toxic metals. Evidence supporting this hypothesis is presented, concentrating on studies that have examined the distribution of toxic metals in the human nervous system. Clinicopathological features shared between neurological disorders are listed that can be linked to toxic metals. Details are provided on how the hypothesis applies to multiple sclerosis and the major neurodegenerative disorders. Further avenues to explore the toxic metal hypothesis for neurological disorders are suggested. In conclusion, environmental toxic metals may play a part in several common neurological disorders. While further evidence to support this hypothesis is needed, to protect the nervous system it would be prudent to take steps to reduce environmental toxic metal pollution from industrial, mining, and manufacturing sources, and from the burning of fossil fuels.

## 1. Introduction

It is widely considered that most of the common sporadic neurological disorders are due to genetic susceptibilities that are triggered by exposure to environmental agents (1). The phrase often heard is that “your genes load the gun; the environment pulls the trigger.” Whole genome and exome analyses of large numbers of people affected by neurological diseases, as well as non-affected controls, have revealed a multitude of genetic variants that predispose to either multiple sclerosis (MS) (2) or the common neurodegenerative disorders amyotrophic lateral sclerosis (ALS) (3), Parkinson disease (PD) (4) and Alzheimer disease (AD) (5). Finding environmental triggers for these diseases, however, has proven more difficult. Although the human genome is large, it does have limits, whereas the scope of potentially damaging environmental agents appears virtually limitless. Epidemiological investigations have come up with several candidate environmental toxic agents (toxicants) based on occupational exposures, such as artisanal gold miners using mercury (6), but most people with neurological diseases do not work in these occupations and are in fact usually unaware of what toxicants they may have been exposed to throughout their lives. Many newly-synthetized chemicals appear every year, and mining and fossil fuel industries increasingly pollute the atmosphere, water, and soil with potential toxicants, making it difficult for researchers to keep up with an ever-changing toxicant landscape (7).

Studies using experimental animals have been of value in assessing the role of candidate toxicants in neurological disorders, in particular showing how low levels of toxicant exposure can lead to nervous system uptake (8), which cells contain the toxicants (9), how toxicants enter the nervous system (10), how metal toxicants can pass through the placenta and enter the fetal nervous system (11), and how combinations of toxicants have synergistic deleterious actions on nervous tissue function (12). However, it is difficult for experimental animal studies to re-create the lifetime of exposure to multiple toxicants that is experienced by humans with their different propensities to take up toxicants into the brain and their unique genetic variations. Moreover, studies on experimental animals are generally performed on young animals with short life spans, while AD, PD and other neurodegenerative conditions are typically disorders of later ages (13).

Examination of autopsied brains from people who have died with neurodegenerative diseases have given important clues for possible avenues of research to pursue (14). However, a difficulty in brain toxicant research is that most patients with neurological conditions live many years after symptoms of their diseases appear, during which time they have extensive losses of brain cells, often of the cells most likely to be affected by toxicants, with subsequent astrocytic scarring. The cells then remaining for study at autopsy are likely to be those that were not affected by the toxicants, a “survivor effect.” Damaged cells can also secondarily take up metal toxicants (mineralization) which are not relevant to the initial pathogenesis. Examining autopsied tissue to look for toxicant-related causes of a neurological disorder can therefore be compared to trying to discover the cause of a war by examining the body of a dead soldier on the battlefield. One way around this problem is to look instead at the distribution of toxicants within the brains of people without neurological disorders, who may not have developed a disorder because toxicants had not accumulated to a critical level, or who did not have deleterious genetic variants creating susceptibility to such disorders.

The effects of toxicants on the brain vary according to the age (pre-natal, perinatal, youth, and older) of the individual, and the dose, duration, and frequency of exposure (7). Toxicants can damage neurons without causing neuronal cell body loss (15), so just because neuronal numbers are normal does not mean that the cells have not been injured by a toxicant. Furthermore, the brain has efficient mechanisms of clearing toxicants from cells (16, 17), so a cell not containing toxicants can still have impaired function, a “hit and run” situation. This means that when looking for toxicants in human brains, large numbers of participants are required, since the relevant toxicant may only be observable if the toxicant exposure was recent or ongoing. An exception to this is the human locus ceruleus in the brain stem, which appears to selectively take up and retain metal toxicants for long periods of time (18–26). Therefore, examining the locus ceruleus can indicate previous exposures to toxicants that may have been cleared from other regions of the brain after permanently affecting cell function.

In this article, the terms “toxic metals” or “metal toxicants” are used for the toxicants we have studied, though a more comprehensive term that covers the full range of possible environmental toxicants is “potentially toxic elements” (27). This is because not all environmental toxicants that can affect the brain are metals. In addition, many non-essential metals that are potentially toxic can be found in the brains of people without neurological disorders since they have been effectively neutralized by binding to agents such as selenium or metallothionein or they are safely ensconced within lysosomes. Finally, some essential metals, like iron (28), can be toxic if there is too much of them, or if they react with other toxic metals. Since our work has studied metal toxicants, however, here we shall refer to them by the shortened nomenclature above. In this article the term “neurological disorders” refers to the four conditions considered in this hypothesis, i.e., MS, ALS, PD and AD. The term “neurodegenerative disorders” refers to ALS, PD, and AD.

Toxicants usually appear in only small numbers of cells of the brain, so one cannot use bulk chemistry to detect them due to the dilution effect of the non-toxicant containing cells. Fortunately, there are elemental bioimaging methods that can get around this problem, such as neutron activation analysis that can measure many metals with high sensitivity and specificity (29). Another method is autometallography (AMG), a silver-based technique that can detect very small amounts of inorganic mercury, silver, and bismuth within cells (30, 31). AMG can be supplemented by laser ablation-inductively coupled-mass spectrometry imaging (LA-ICP-MSI) which can detect a large array of elements within cells and tissues, though at a lower resolution than autometallography (26). Using AMG and LA-ICP-MSI together gives a good picture of toxicant distribution within human brains. Another technique, synchrotron X-ray fluorescence microscopy, gives excellent cellular resolution for multiple elements (24), but requires frozen tissue and very small sample sizes, making it unsuitable for large-scale human brain-based projects.

Having access to large numbers of human brains, from people both with and without neurological conditions, allows for the comparison of toxicants in normal and damaged brains. Looking at the distribution of toxic metals in these brains using elemental bioimaging techniques has provided background data for a hypothesis that toxic metals play a role in triggering common human neurological disorders. In the following sections, we will summarize the data that led up this hypothesis, examine similarities between the major neurological disorders that could be due to toxic metals, show how the hypothesis may be applied to MS, ALS, PD, and AD, and offer suggestions for future experiments that might (or might not) support this hypothesis.

## 2. The toxic metal hypothesis

**Figure 1:**
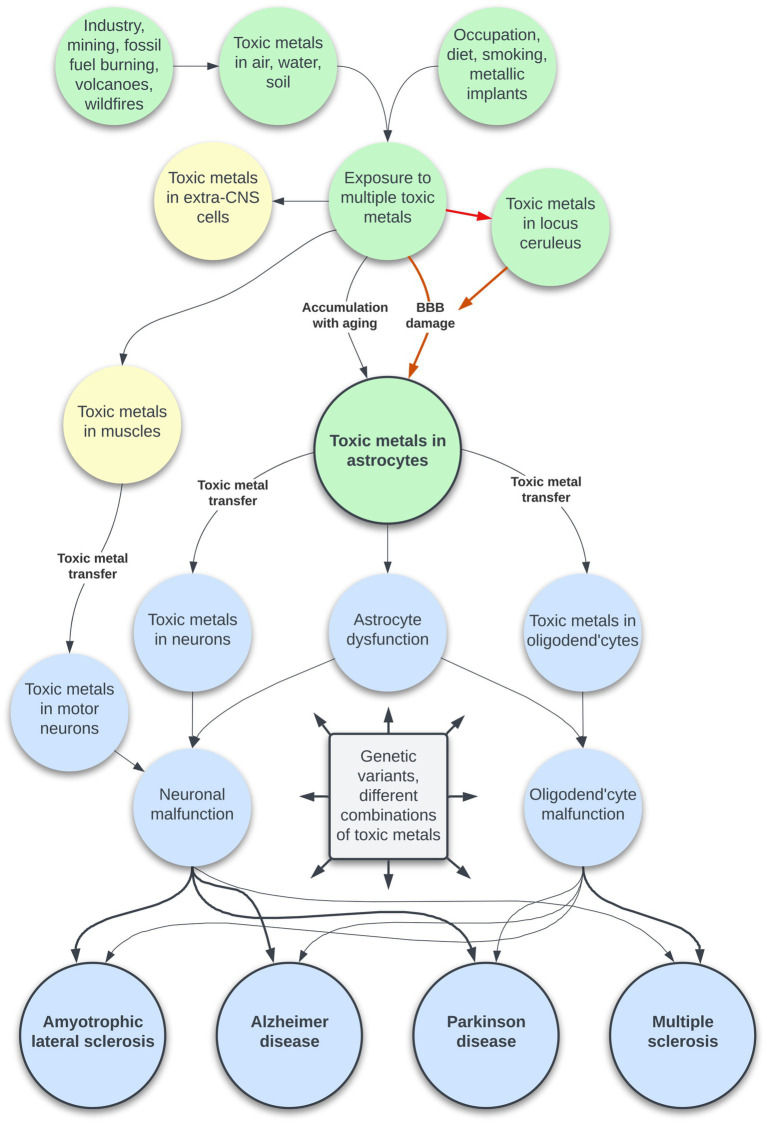
The toxic metal hypothesis for neurological disorders. Human exposure to multiple toxic metals results in selective uptake of the metals into locus ceruleus neurons. Decreased noradrenaline from the locus ceruleus causes multifocal damage to the blood–brain barrier, allowing toxic metal uptake by astrocytes. A second pathway is slower age-related accumulation of toxic metals through an intact blood–brain barrier. Toxic metals in astrocytes cause astrocyte dysfunction, and transfer of metals from astrocytes triggers neuronal and oligodendrocyte malfunction. The degree of toxic metal-induced damage to these cells differs due to genetic variants and different combinations of toxic metals, leading to varying clinical outcomes. Uptake of toxic metals by extra-CNS organs is responsible for systemic disorders associated with neurological disorders. Uptake of toxic metals by striated muscles is followed by retrograde transport of metals to the lower motor neurons affected by ALS.

The toxic metal hypothesis for neurological disorders (Figure 1) is that: (i) Environmental toxic metals are taken up by neuromelanin-containing locus ceruleus neurons, leading to multifocal damage to the blood–brain barrier. (ii) Circulating toxicants then enter astrocytes which transfer the toxic metals to oligodendrocytes and other neurons. (iii) Different neurological disorders result from variations in the degree of dysfunction of toxic metal-containing astrocytes, oligodendrocytes, and neurons, based on genetic variants and different combinations of toxic metals.

## 3. Evidence for the toxic metal hypothesis

This review of evidence for the toxic metal hypothesis will concentrate on studies of the distribution of toxic metals in the human brain. Where human autopsy material is not suitable for examination, for example for subcellular distributions of metals that require electron microscopy, animal studies will be cited. Reviews of the environmental sources, epidemiology and experimental toxicity of toxic metals in relation to neurological disorders will not be presented since these are available elsewhere (32). For studies that have used autometallography to detect inorganic mercury, silver, and bismuth, the three metals are referred to as “autometallography-detected toxic metals” (AMG™). Previous exposures to toxic metals are inferred from the elements detected in the locus ceruleus by LA-ICP-MSI.

### 3.1. People exposed to toxic metals

Although an autopsy series of many people who did not have known neurological disorders has shown on autometallography that previous exposures to AMG™ are common, it is unusual to be able to find a known source of toxic metal exposure from clinical records. However, in one person in our autopsy series a continuous exposure to inorganic mercury was known, in another intermittent exposure to organic mercury was likely, and in a third exposure to silver from an unknown source could be inferred by LA-ICP-MSI findings, as detailed below.

#### 3.1.1. Continuous exposure to elemental mercury

**Figure 2:**
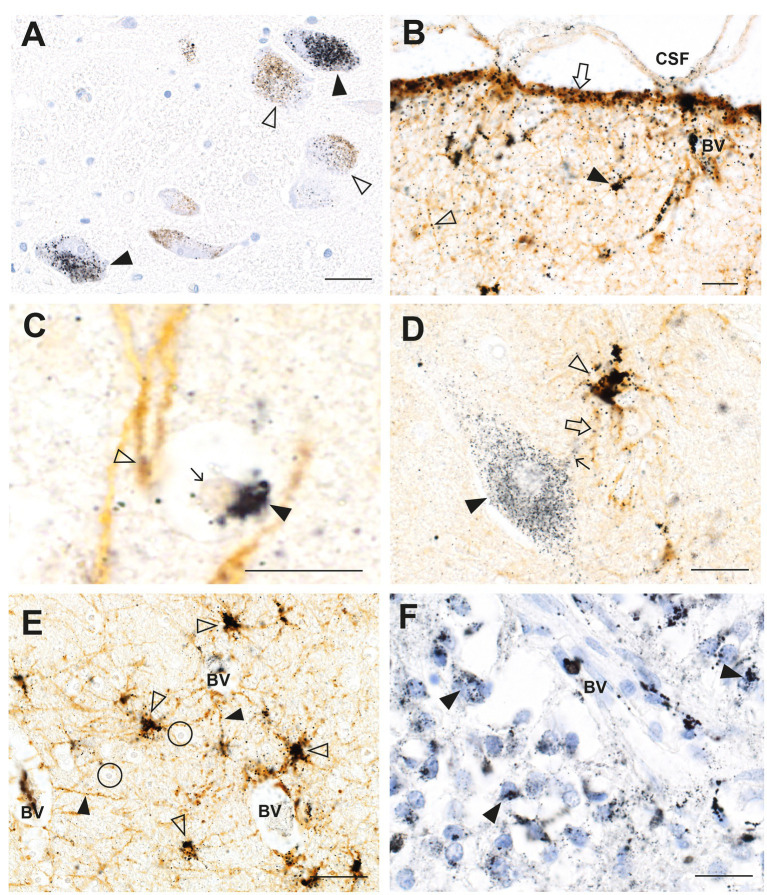
Mercury in the brain after exposure to inorganic mercury. **(A)** Dense black AMG™ staining is seen in two locus ceruleus neurons (closed arrowheads) and scattered in the neuromelanin pigment in another two neurons (open arrowheads). **(B)** In the frontal cortex, AMG™ (combined with brown GFAP immunostaining) are present in the frontal lobe brown glia limitans (arrow) below the cerebrospinal fluid (CSF), in brown subpial astrocyte cell bodies (closed arrowhead), in brown astrocyte processes (open arrowhead), and in blood vessel walls. **(C)** In the frontal cortex, AMG™ (closed arrowhead) are seen in an oligodendrocyte cell body adjacent to the nucleus (thin arrow). The GFAP-stained brown descending branch of an interlaminar astrocyte makes contact (open arrowhead) with the oligodendrocyte. **(D)** In the frontal cortex, AMG™ are seen in the cell body of a corticomotoneuron (closed arrowhead), a dendrite (small arrow), a GFAP-stained brown connecting astrocyte process (large arrow), and largely obscuring the brown astrocyte cell body (open arrowhead). **(E)** In the frontal white matter, GFAP-stained brown fibrous astrocyte cell bodies (open arrowheads) and processes (closed arrowheads) contain AMG™. No AMG™ are seen in oligodendrocytes (in circles). **(F)** In the pineal gland, AMG™ are present in most pinealocytes (closed arrowheads) and in blood vessel walls. BV blood vessel. Methods: **A–E** adapted from (23), **F** from (25). Bars = 20 μm.

In a man who had injected himself intravenously with metallic mercury, mercury deposits throughout his body ensured that he had a continuous exposure to mercury for 5 months before his death by suicide (23, 33). Autometallography (Figure 2) detected mercury at high levels in locus ceruleus neurons, in all classes of astrocytes (grey matter subpial, interlaminar, protoplasmic, varicose, and white matter fibrous), grey but not white matter oligodendrocytes, corticomotoneurons, brain post-capillary venule endothelial cells, pinealocytes, renal proximal tubules, and hepatocytes.

#### 3.1.2. Intermittent exposure to organic mercury

**Figure 3:**
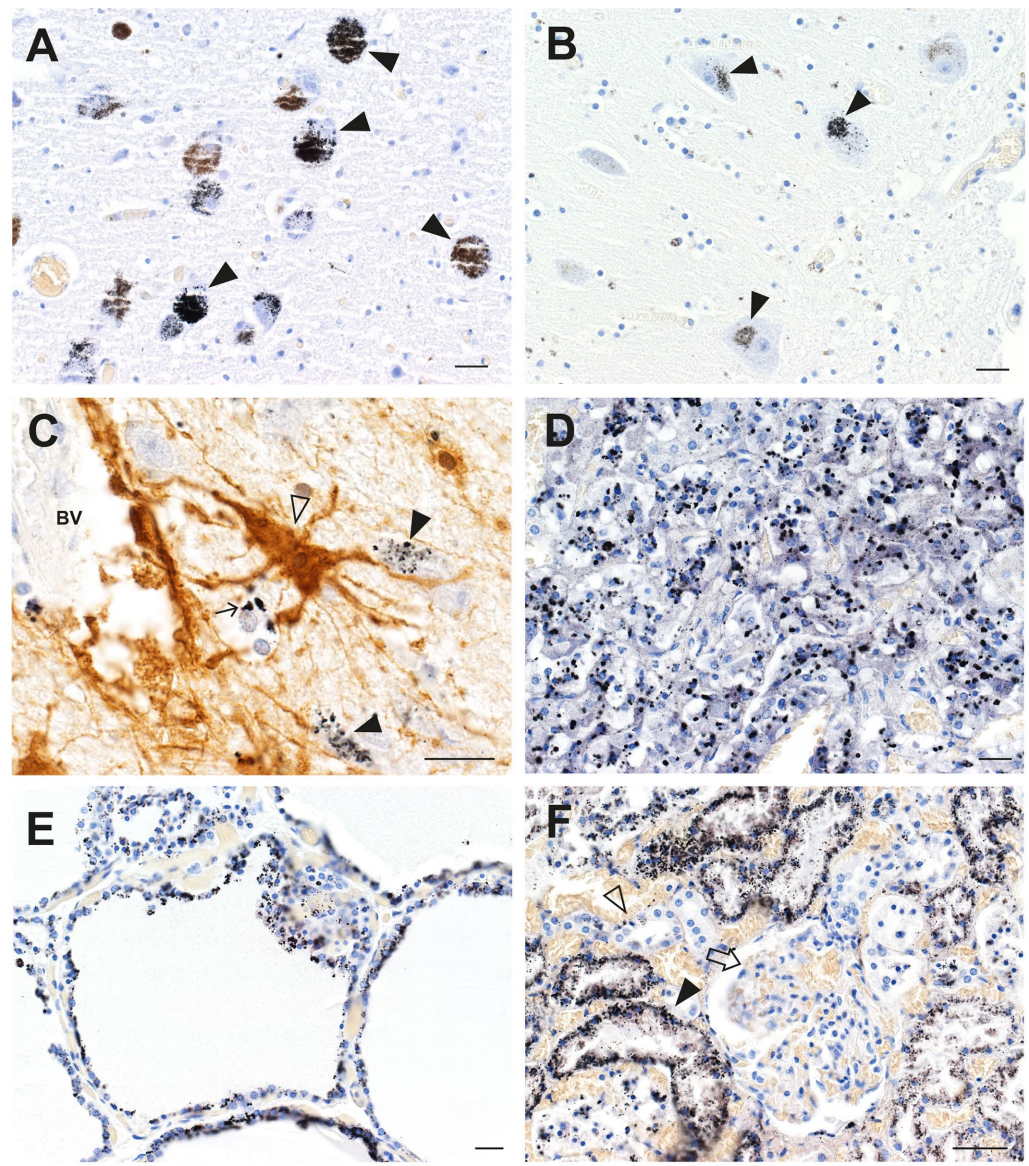
Mercury in brain and other organs after intermittent exposure to organic mercury. **(A)** In the locus ceruleus, dense black AMG™ staining is seen in most neurons (closed arrowheads). **(B)** AMG™ are present in cerebellar dentate neurons (closed arrowheads). **(C)** In the lateral geniculate nucleus, AMG™ staining is seen in a GFAP-immunostained brown perivascular astrocyte (open arrowhead), in nearby neurons (closed arrowheads), and in an oligodendrocyte (small arrow). **(D)** Many chromaffin cells in the adrenal medulla contain AMG™. **(E)** Most thyroid follicular cells contain AMG™. **(F)** Renal proximal tubule cells contain AMG™ (closed arrowhead), but not distal tubules (open arrowhead) or glomeruli (arrow). BV blood vessel. Methods: **A** (22), **B** (26), **C** adapted from (25), **D** (34), **E** (35), **F** (36). Bars = 20 μm.

An autopsy was performed on a professional fisherman who drowned at sea. Autometallography showed widespread mercury (confirmed on LA-ICP-MSI) in cells of his brain and general organs (Figure 3), presumably because of a high dietary intake of methylmercury in fish. In the brain, neurons most affected were those in the locus ceruleus, the cerebellar dentate nuclei, and the lateral geniculate nuclei. Many perivascular astrocytes and oligodendrocytes contained mercury (25). In the general organs, most mercury was seen in adrenal medulla chromaffin cells, renal proximal tubules and Henle loops, thyroid follicular cells, and pancreatic islets.

#### 3.1.3. Exposure to silver from an unknown source

**Figure 4:**
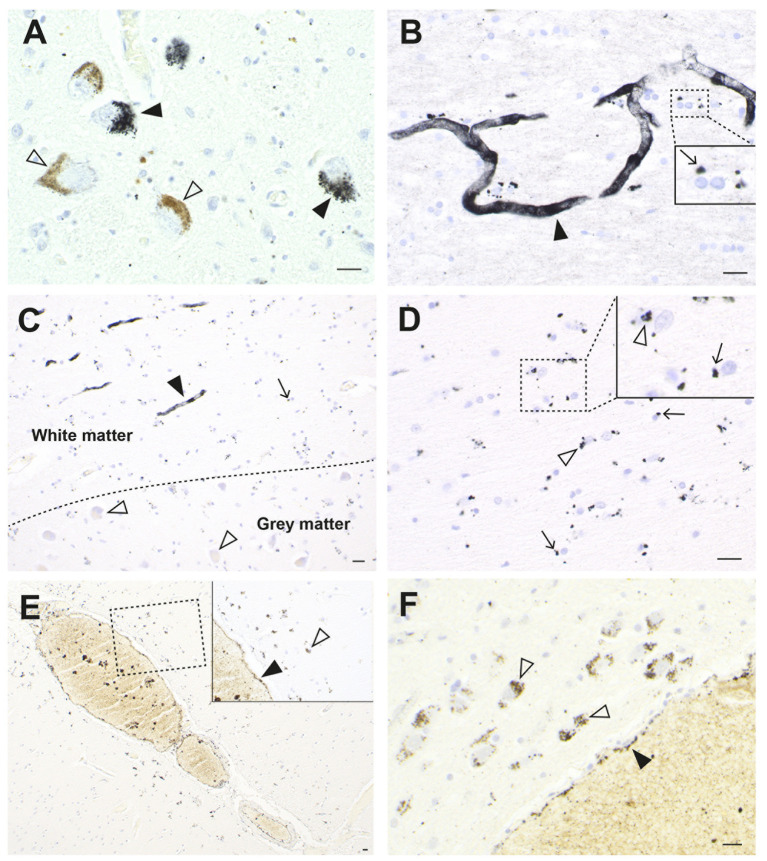
Silver in the brain from a man with an unknown source of exposure. **(A)** In the locus ceruleus, dense black AMG™ staining is seen in two neurons (closed arrowheads). Another two neurons are AMG™-free (open arrowheads). **(B)** In the anterior pontine white matter, AMG™ staining is present in the walls of a branching microvessel. In the magnified box (at right) paranuclear AMG™ deposits (thin arrow) are seen in oligodendrocytes. **(C)** AMG™ are prominent in microvessels (closed arrowhead) and in glial cells (small arrow) in the inferior olive white matter in the medulla oblongata. No AMG™ are present in blood vessels, glial cells, or neurons (open arrowheads) in the grey matter of the adjacent inferior olivary nucleus. **(D)** In the inferior olive white matter in the medulla oblongata, AMG™ are present in the cytoplasm of astrocytes (open arrowheads) and oligodendrocytes (thin arrows). **(E)** AMG™ are present in endothelial cells (closed arrowhead) of a venule deep in the lateral occipitotemporal gyrus of the inferomedial temporal lobe, and in small superficial cortical cells adjacent to the venule (open arrowhead). Inset is a magnification of the dashed rectangle. **(F)** A venule (bottom right) in the anterior pons has AMG™ in its endothelial cells (closed arrowhead). Adjacent pontine neurons (open arrowheads) contain AMG™. Methods: **A,E,F** (26), **B–D** adapted from (26). Bars = 20 μm.

When sections from the brain of a man who died suddenly and unexpectedly were examined with autometallography, silver (confirmed on LA-ICP-MSI) was found in widespread brain regions (26). Cells containing most silver were locus ceruleus neurons, white (not grey) matter oligodendrocytes, blood vessel walls in the anterior pons and lateral geniculate nuclei (with less staining in microvessels in white matter elsewhere), white matter astrocytes, cerebellar dentate neurons, brain stem raphe neurons, ependymal cells, and neurons and glial cells adjacent to medium-sized venules in the leptomeninges, especially those deep in the lateral occipitotemporal gyrus of the inferomedial temporal lobe (Figure 4).

These three cases show how exposure to different types of toxic metals (elemental mercury, organic mercury, and silver) can result in some overlap in their distribution in the brain (e.g., all present in the locus ceruleus), but that different regions and cells of the brain can be affected (e.g., either grey or white matter oligodendrocytes). Factors underlying these differences are likely to include differences in genetic susceptibility to the toxicants, the degree and frequency of toxicant exposure, and various combinations of synergistic toxic metals. These differences may underlie the reason exposures to toxic metals can result in different neurological disorders.

### 3.2. Shared clinicopathological features of MS, ALS, PD, and AD, and toxic metal links

While the core clinicopathological features of MS, ALS, PD, and AD are distinctive enough to allow accurate diagnostic categorization, they share several clinicopathological features (Table 1). The toxic metal hypothesis proposes that metal toxicants will be able to account for these shared features. The references in Table 1 to potential toxic metal links are mostly related to the metals detectable on autometallography, such as mercury.

**Table 1:** Shared clinicopathological features of MS, ALS, PD, and AD, with toxic metal links.

		MS	ALS	PD	AD	Toxic metals
Clinical	Incidence	Increasing (37)	Increasing (38)	Increasing (39)	Decreasing? (40)	Increasing in atmosphere (41), sea (42)
	Typical age of onset	20–35 y (2)	60–79 y (3)	65 + y (4)	65 + y (5)	Accumulate in cells with aging (43)
	Progression	Yes (2)	Yes (3)	Yes (4)	Yes (5)	Inorganic forms accumulate (44)
	Sleep disorder	Yes (2)	Yes (3)	Yes (4)	Yes (5)	In pineal, suprachiasmatic nucleus (45)
	Smoking risk factor	Yes (2)	Yes (3)	No? (4)	Yes (46)	In cigarette smoke (47, 48)
	Extra-CNS organs	Yes (49)	Yes (50)	Yes (4)	Yes (51)	In extra-CNS organs (25, 34–36, 52–56)
	Metabolic changes	Yes (57)	Yes (3, 58)	Yes (59)	Yes (51, 59)	In endocrine organs (34, 35, 53, 55)
	Hallucinations	Yes (60)	Yes (61)	Yes (62)	Yes (63)	In geniculate nuclei (64)
Cells	Selective neurons	Involved (2)	Involved (3)	Involved (4)	Involved (5)	In selective neurons (18, 23, 25, 26, 33, 65)
	Locus ceruleus	Involved (66)	Involved (67)	Involved (68, 69)	Involved (70)	In locus ceruleus neurons (19–21, 24)
	Astrocytes	Involved (71)	Involved (72, 73)	Involved (67)	Involved (5, 73–75)	In astrocytes (23, 64, 76)
	Oligodendrocytes	Involved (2)	Involved (77, 78)	Involved (79)	Involved (74)	In oligodendrocytes (23, 25, 26, 64)
	Endothelial cells	Involved (2)	Involved (80)	Involved (81)	Involved (82)	In endothelial cells (56, 64)
	Pericytes	Involved (83)	Involved (84)	Involved (81)	Involved (82, 85)	In pericytes (26)
	Retinal	Involved (86)	Involved (87)	Involved (88)	Involved (89)	In retina (54, 90)
	Blood–brain barrier	Damaged (2)	Damaged (84)	Damaged (81)	Damaged (82, 85)	Damage blood–brain barrier (91)
Subcellular	Mitochondria	Involved (2)	Involved (3)	Involved (4)	Involved (92)	In mitochondria (93)
	Lysosomes	Involved (94)	Involved (94–96)	Involved (4, 94)	Involved (94)	In lysosomes (9)
Mechanisms	Inflammation	Yes (2)	Yes (3)	Yes (4)	Yes (5, 97)	Promote inflammation (98)
	Autoimmunity	Yes (2, 99)	Yes (3, 100)	Yes (4, 101)	Yes (102)	Promote autoimmunity (98)
	Oxidative stress	Yes (2)	Yes (103)	Yes (104)	Yes (92, 105)	Promote oxidative stress (106, 107)
	Apoptosis	Yes (108)	Yes (109)	Yes (110)	Yes (111)	Promote apoptosis (112)
	Protein aggregation	Yes (113)	Yes (3)	Yes (4)	Yes (5)	Promote protein aggregation (114, 115)
	Glymphatic pathway	Involved (116)	Involved (117)	Involved (118)	Involved (5)	In astrocytes (23, 26, 64)
Genetics	Somatic mutations	Yes (119)	Yes (120–125)	Yes (125–128)	Yes (125, 129, 130)	Affect DNA (131–133)
	DNA methylation	Yes (134, 135)	Yes (136, 137)	Yes (138)	Yes (139)	Affect DNA methylation (140–142)
	Germline variants	Yes (2)	Yes (3)	Yes (4)	Yes (5)	Toxicity modified (143)

CNS, central nervous system.

#### 3.2.1. Clinical features

Shared clinical features of MS, ALS, PD and AD include an increasing disease incidence over time. The data for this are strongest for MS (37) and PD (39) and moderate for ALS (38). The incidence of dementia overall appears to be decreasing in some studies (40), though studies specifically for AD are ongoing. These increases in disease incidence could be related to the fact that levels of toxicants such as mercury are increasing steadily in the atmosphere, and hence into water and fish, probably from the burning of fossil fuels (41) and from artisanal mining which uses mercury for gold extraction (6).

While aging is the major risk factor for neurodegenerative diseases such as AD and PD, it remains unclear which of the biological hallmarks of aging are relevant to the pathogenesis of these disorders (144). The older adult age of clinical onset of the neurodegenerative disorders could be attributed to toxic metals slowly accumulating within permanent cells of the brain until a critical level is reached that causes cell damage. Toxic metals increase in locus ceruleus neurons during aging, until a fall in numbers in late older age (43), the latter probably because of a survivor effect from those people who have been exposed to fewer metal toxicants during their lives. People with MS usually present at an earlier age than those with neurogenerative disorders, possibly because their genetic propensity to autoimmunity (2) makes them susceptible early in life to the autoimmune effects of toxic metals (98).

All four neurological disorders under consideration have progressive phases, though MS is usually remitting–relapsing in its early stages (2). Exposure to toxic metals such as mercury is often from organic forms (such as from consumption of predatory fish such as sharks) which can pass through the blood–brain barrier and enter the brain (145). Organic mercury is slowly converted to more toxic inorganic mercury in the brain (44), which could be behind the slow progression of neurodegenerative diseases. Furthermore, toxic metals can cause protein conformational changes (114, 115), which could then slowly spread the disorder to other connected cells (121).

Sleep disorders are recognized as a part of the neurological disorders listed in Table 1 (2–5). In a man who injected himself with metallic mercury (23, 33), most cells in the pineal gland contained mercury (Figure 2F), probably because the pineal, important for sleep regulation, is outside the blood–brain barrier. Another region involved in sleep regulation, the suprachiasmatic nucleus, has been found in rodents to take up bismuth selectively (45). Bismuth and mercury have similar distributions in the brain (146), suggesting other toxic metals could target the suprachiasmatic nucleus. The suprachiasmatic nucleus is rarely seen in routinely autopsied human brains, and the brain must be removed and dissected specifically to locate this nucleus.

Smoking has been proposed to be a risk factor for MS (2), ALS (3), and AD (46), but possibly not for PD (46). Cigarette smoke contains several toxic metals, especially cadmium and mercury (47, 48), and so could increase the toxic metal burden within brain cells.

Cell degeneration in neurological disorders is non-autonomous, since neurons and glial cells are both affected in most disorders (147). It has also been noticed that non-CNS organs are often involved in neurological disorders (4, 49–51). Toxic metals are usually found in multiple CNS and non-CNS organs within the same individuals (43). Many of these are endocrine organs such as the thyroid, pituitary, adrenal medulla, and pancreatic islets (34, 35, 53, 55), which could account for metabolic changes such as hyperenergetic states, due to mercury in the adrenal medulla overproducing noradrenaline (34), and endocrine deficiencies. Hypertension associated with neurological disorders could result from toxicant-induced kidney damage (36), and cardiovascular problems could arise from toxicants in endothelial cells of non-CNS blood vessels (56). Current hypotheses for the spread of cell damage in neurodegenerative conditions, such as a prion-like spread of aggregated protein, are not able to explain these extra-CNS phenomena, whereas toxic metals can account for both CNS and associated non-CNS disorders.

Visual and auditory hallucinations may be present in all four neurological disorders (60–63), most prominently in PD and AD. Hallucinations can be caused by interference with the transmission of sensory signals along visual and auditory pathways (148). Toxic metals have a predilection to be taken up by the lateral geniculate nucleus (on the visual pathway) and by the medial geniculate nucleus (on the auditory pathway) (64), so impaired function of these two nuclei could contribute to hallucinations in toxicant-associated neurological disorders.

#### 3.2.2. Affected cells

Not all CNS cells are involved in neurological disorders, and different toxic metals also affect only some types of cells in restricted regions of the nervous system (18, 23, 25, 26, 33, 65). A site where many neurons contain toxic metals is the neuromelanin-containing locus ceruleus (22, 149), though why only certain locus ceruleus neurons contain these metals, often adjacent to neurons without metals, is unknown. The locus ceruleus can contain at least five different toxic metals in one person (see below). The widespread noradrenergic output of the locus ceruleus has multiple protective effects on neurons, glial cells, and the blood–brain barrier (150), so focal damage to locus ceruleus neurons from toxic metals could underlie the multifocal cell damage described in neurological disorders.

The other major region of the brain that harbors neuromelanin-containing neurons is the substantia nigra, whose neurons also bind and accumulate large amounts of toxic metals such as mercury and cadmium to form stable and insoluble complexes that remain inside the neurons for long periods of time (29, 149). The later section on PD will address this issue in greater detail.

Other CNS neurons commonly containing toxic metals are those in the thalamus, cerebellar dentate nuclei, medullary olivary nuclei, brain stem midline raphe, corticomotoneurons, spinal motor neurons, and spinal inhibitory interneurons (18, 23, 25, 26, 33, 65).

Glial and endothelial cells implicated in the pathogenesis of neurological disorders often contain toxic metals. As mentioned, all five types of astrocytes can contain AMG™ (23). Perivascular astrocytes have the greatest chance of containing AMG™, which can be seen in their cell bodies and processes, and in nearby oligodendrocytes and neurons (Figure 3C). In any microscopic field, usually only some astrocytes contain toxic metals. Small perinuclear deposits of AMG™ are often seen in oligodendrocytes, either in grey matter (Figure 2C) or white matter (Figure 4D). Endothelial cells of microvessels may contain AMG™, especially those in the white matter (Figure 4C), or in the anterior pons (Figure 4B) and geniculate nuclei. Pericytes adjacent to endothelial cells can also have AMG™ in their cytoplasm (26) (see below), would may be of importance since pericyte dysfunction has been implicated in several neurological disorders (81, 83, 84).

Thinning of the retina has been described as an early feature in several neurological disorders (see Table 1) (86–89). Metals may play a part in this retinal thinning, since an LA-ICP-MSI study has shown several toxic metals in the retina and the optic nerve head of humans unaffected by neurological disease (54), and transplacental mercury can be taken up by the retina and optic nerve of fetal mice (90).

Early damage to the blood–brain barrier appears in all four neurological disorders under consideration (Table 1) (2, 81, 82, 84, 85). Toxic metals can affect the blood–brain barrier in two ways, by directly impairing the function of toxicant-containing endothelial cells and pericytes, or by decreasing the amount of protective noradrenaline from toxicant-damaged locus ceruleus neurons (151). Mercury, for example, is one toxic metal suspected to cause vascular dysfunction because of its effects on endothelial cells and the blood–brain barrier (91).

No AMG™ staining has been detected in microglia in neurodegenerative disorders in our studies, using immunohistochemical stains for microglia combined with autometallography (152). This suggests that microglial activation in toxicant-induced neurodegenerative disorders is occurring downstream from the toxic actions of the metals and is not a primary driver of disease. On the other hand, some macrophages containing AMG™ have been seen in MS perivascular spaces (26) (see below) and could be contributing to the disordered immune response in MS.

#### 3.2.3. Affected subcellular organelles

Mitochondria (2–4) and lysosomes (4, 94–96) are thought to be involved in many neurological diseases. The contents of subcellular organelles need to be visualized by electron microscopy, but tissue preservation of routinely autopsied brain tissue is inadequate for electron microscopy. However, animal experimentation combining autometallography and electron microscopy has shown that mercury binds preferentially to membranous organelles in the cell, including mitochondria, endoplasmic reticulum, the Golgi apparatus, and the nuclear membrane (93), all of which have been implicated in neurological disorders. Lysosomes usually safely sequester intracellular toxic metals such as mercury (9). However, a toxic metal overload could cause lysosomal damage, with widespread deleterious consequences for the cell (153).

#### 3.2.4. Pathological mechanisms

Most pathological mechanisms thought to underlie cell loss in neurological disorders (Table 1) could be triggered by toxic metals, since these metals promote inflammation and autoimmunity (98), oxidative stress (106, 107), apoptosis (112), and protein aggregation (114, 115). The dysfunction of the glymphatic toxicant clearance pathway described in neurological disorders (3, 5, 116) could be due to toxic metals, since astrocytes, a key player in the glymphatic pathway (17), often contain large amounts of AMG™ (23, 26, 64) which could affect astrocyte function (154).

#### 3.2.5. Genetic changes

Somatic mutations, either in nuclei or mitochondria, have been hypothesized to play a part in several neurological disorders (122), and this concept has support from studies in MS (119), ALS (120–125), PD (125–128) and AD (125, 129, 130). Toxic metals can cause genetic damage (131–133), and the widespread presence of these metals within cells of the nervous system suggests they could be triggers for somatic mutations which then spread damage to other connected cells, possibly by misfolded proteins (121).

Epigenetic changes to DNA methylation for gene silencing have been proposed to be involved in MS (134, 135), ALS (136, 137), PD (138), and AD (139), so it may be relevant that toxic metals such as mercury can cause epigenetic alterations (140–142).

Numerous susceptibility variants have been uncovered for the sporadic neurological disorders (2–5). Many genetic variants also influence how humans deal with metal toxicants (143). Some of these variants may overlap, with the susceptibility to toxic metal damage leading to the neurological disorders.

### 3.3. The toxic metal hypothesis in MS, ALS, PD, and AD

To see if the toxic metal hypothesis stands up regarding common neurological disorders, one can examine the distribution of toxic metals in the brains of people with these disorders, compared to non-neurologically affected controls, and assess if the presence of toxic metals accords with their clinicopathological features.

#### 3.3.1. Multiple sclerosis

**Figure 5:**
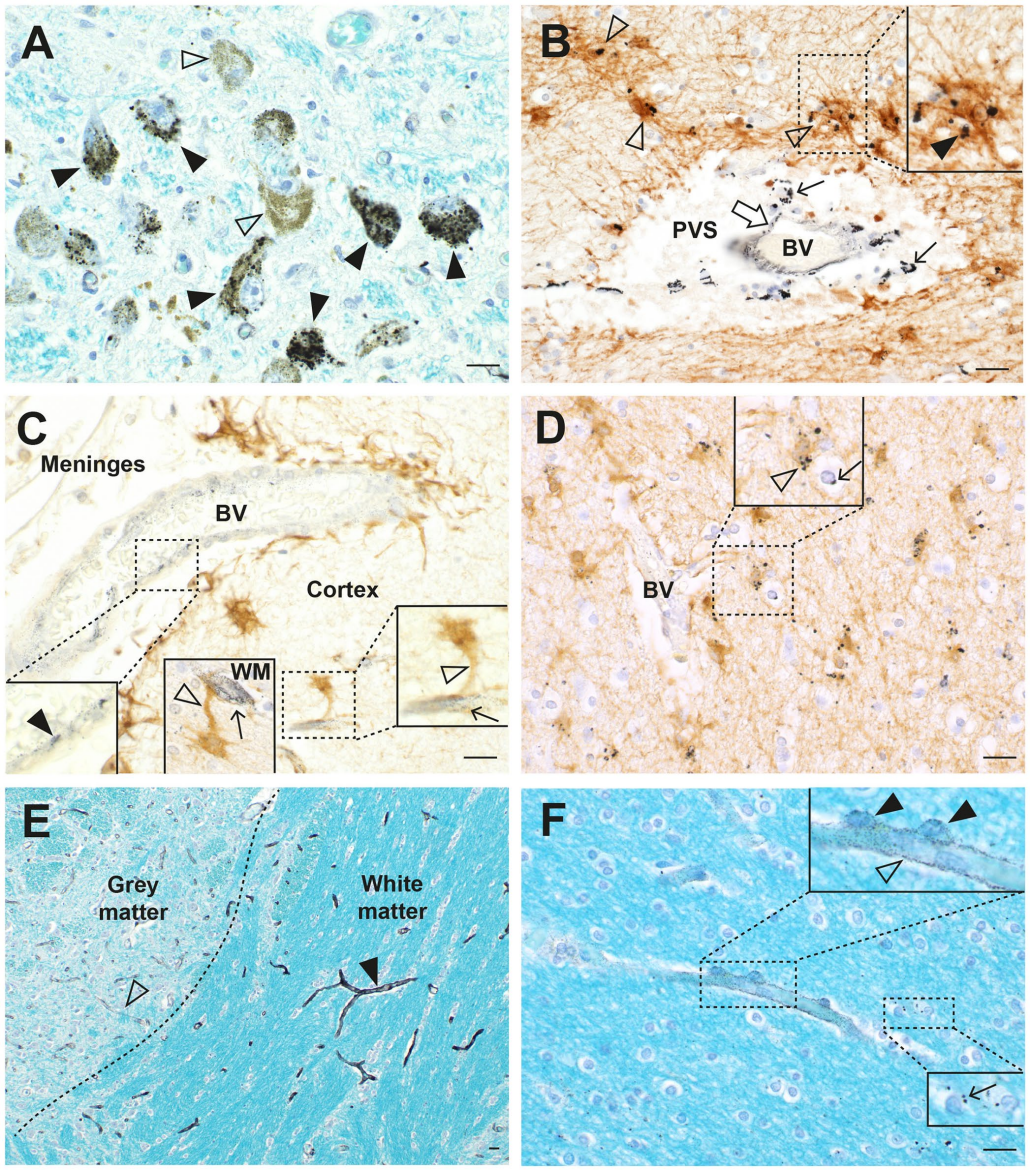
Toxic metals in MS brains. **(A)** In the locus ceruleus, black AMG™ grains are seen in most neurons (closed arrowheads). Some neurons contain no AMG™ (open arrowheads). **(B)** In the pontine white matter, small AMG™ deposits are seen in the wall of a blood vessel (open arrow) and in surrounding macrophages (thin arrows) in the perivascular space (PVS). GFAP-immunostained brown fibrous astrocytes (open arrowheads) near the blood vessel contain AMG™. Inset: magnified view of an astrocyte containing AMG™ (closed arrowhead). **(C)** In the cerebral cortex, a small leptomeningeal blood vessel (BV) contains AMG™ in its wall (left magnified inset, closed arrowhead). Right magnified inset: a microvessel within the cortex contains AMG™ (arrow), with an adjacent GFAP-stained brown astrocyte cell body connected *via* a hypertrophic process (open arrowhead). Middle inset: a white matter (WM) AMG™-containing microvessel (arrow) connects to an astrocyte cell body *via* a hypertrophic astrocytic process (open arrowhead). **(D)** In the cerebral white matter, oligodendrocytes (arrow) and GFAP-stained brown astrocytes (open arrowhead) adjacent to a blood vessel contain AMG™ (magnified in inset). **(E)** Pontine white matter microvessels (closed arrowhead) contain more AMG™ than grey matter microvessels (open arrowhead). **(F)** In the frontal white matter, AMG™ are seen in a microvessel wall (open arrowhead) and in surrounding pericytes (closed arrowheads in magnified upper inset). Lower magnified inset: scattered oligodendrocytes have small AMG™ deposits (arrow). BV blood vessel. Methods: **A–F** adapted from (26). Bars = 20 μm.

The distribution of toxic metals in MS and control brains has recently been described (26). Autometallography of paraffin sections from multiple brain regions of 21 MS patients and 109 controls detected AMG™ in locus ceruleus neurons of both groups, and in widespread blood vessels, oligodendrocytes, astrocytes, and neurons of four MS patients and one control (Figure 5). It was thought these patients with widespread toxic metal in their tissues were likely to have been exposed recently to the toxic metals, whereas these metals had been cleared from most cells by other patients in whom exposure had been further in the past.

**Figure 6:**
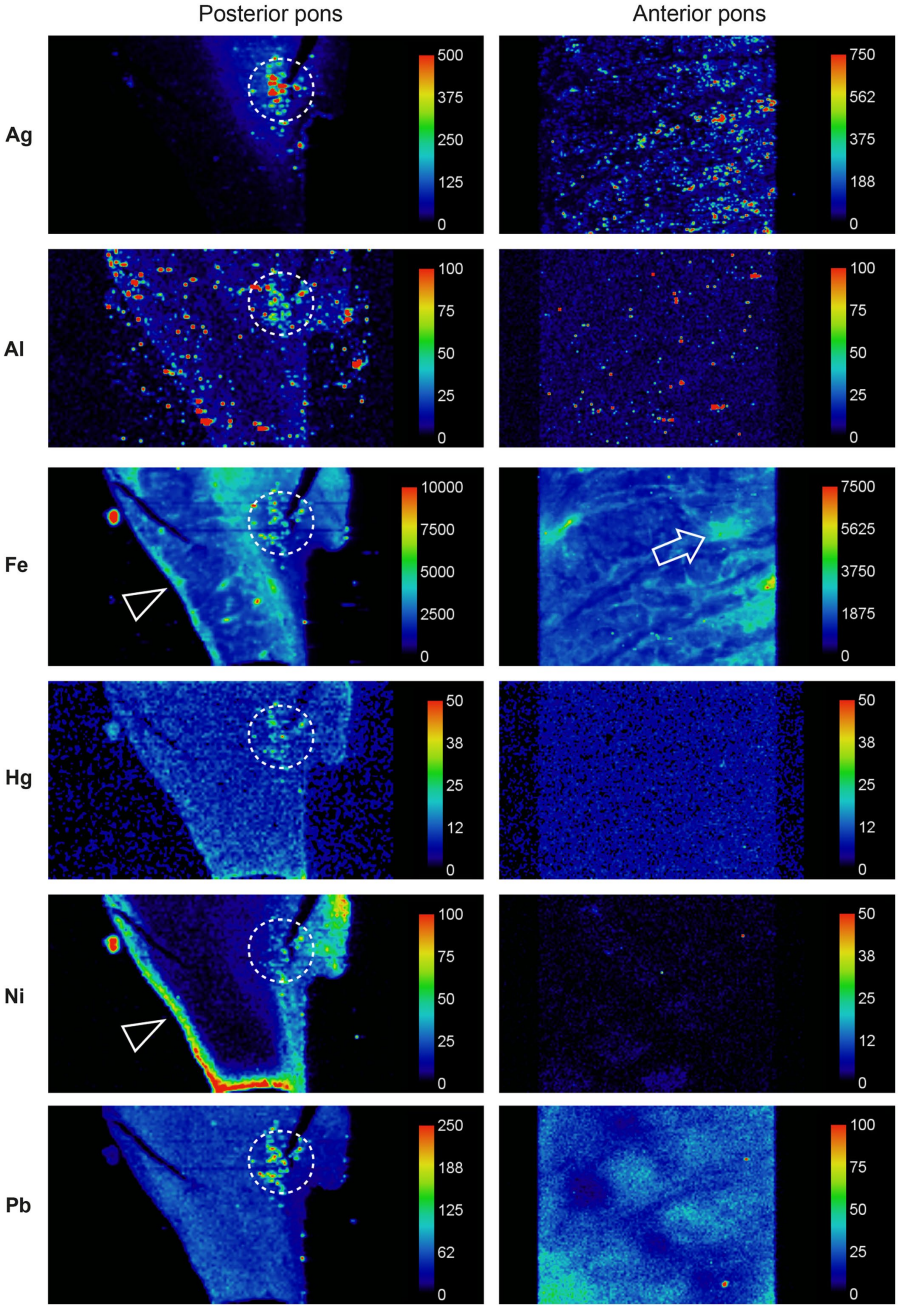
Toxic metals in the pons of a person with MS. *Posterior pons*: metals present in the locus ceruleus (within dashed circles) are silver, aluminium, iron, mercury, nickel, and lead. Iron and nickel are seen in subpial regions adjacent to the cerebrospinal fluid (arrowheads). *Anterior pons*: silver, iron and lead are present in white matter regions, and iron around blood vessels (arrow). Methods: adapted from (26).

LA-ICP-MSI of pons paraffin sections from all MS patients and 12 controls showed that combinations of iron, silver, lead, aluminium, mercury, nickel, and bismuth were present more often in the locus ceruleus of MS patients than controls and were located predominantly in white matter tracts (Figure 6).

Controversy persists as to whether MS results from initial damage to oligodendrocytes, with a secondary immune response (an “inside-out” process), or whether autoimmune-induced inflammation causes oligodendrocyte death (an “outside-in” process) (155). The fact that toxic metals can be found in oligodendrocytes, which already have a high iron content (156), suggests an inside-out mechanism is more likely, with toxic metals promoting apoptosis (108).

**Figure 7:**
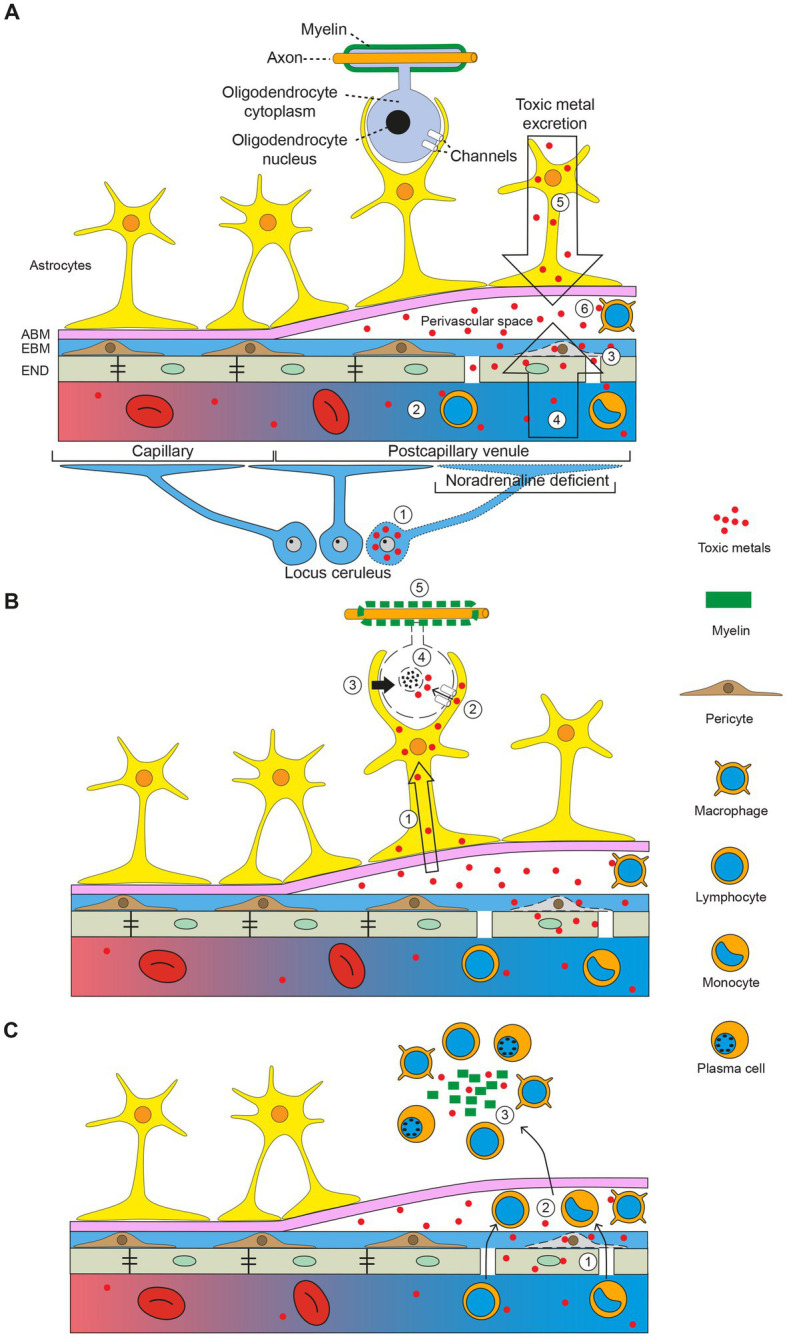
The toxic metal hypothesis in MS. **(A)** Circulating toxic metals enter the perivascular space. (*1*) Toxic metals in locus ceruleus neurons cause a localized noradrenaline deficiency in a postcapillary venule, which impairs the blood–brain barrier. (*2*) Circulating toxic metals activate lymphocytes and monocytes. (*3*) Toxic metals in endothelial cells and pericytes further damage the blood–brain barrier. (*4*) Toxic metals enter the perivascular space from the circulation (first entry). (*5*) Toxic metals exiting the brain via cerebrospinal fluid and astrocytes enter the perivascular space (second entry). (*6*) Toxic metals activate perivascular space macrophages. **(B)** Toxic metals enter astrocytes and oligodendrocytes. (*1*) Toxic metals enter astrocytes from the perivascular space. (*2*) Astrocytes transfer toxic metals into oligodendrocytes through gap junctions. (*3*) Toxic metals activate astrocyte toxicity toward oligodendrocytes. (*4*) Oligodendrocytes undergo apoptosis. (*5*) Demyelination of axons. **(C)** Secondary autoimmune inflammation. (*1*) Activated circulating white blood cells enter the perivascular space. (*2*) White blood cells pass into the brain parenchyma. (*3*) Myelin debris and toxic metals incite an autoimmune inflammatory response. ABM astrocyte basement membrane, EBM endothelial basement membrane, END endothelial cells. Diagram adapted from Mastorakos and McGavern 2019 (157). Reprinted with permission from AAAS.

The demyelinated plaques of MS are usually located around venules, which has prompted studies of reactions in the perivascular space that could give rise to demyelination (157). A potential role for toxic metal uptake in the perivascular space in MS, aided by toxic damage to locus ceruleus neurons impairing the blood–brain barrier, is illustrated in the diagram in Figure 7. This shows how toxic metals can enter the perivascular space, and then enter astrocytes and oligodendrocytes. This triggers oligodendrocyte apoptosis and secondary autoimmune inflammation, with demyelination causing a clinical attack of MS.

**Figure 8:**
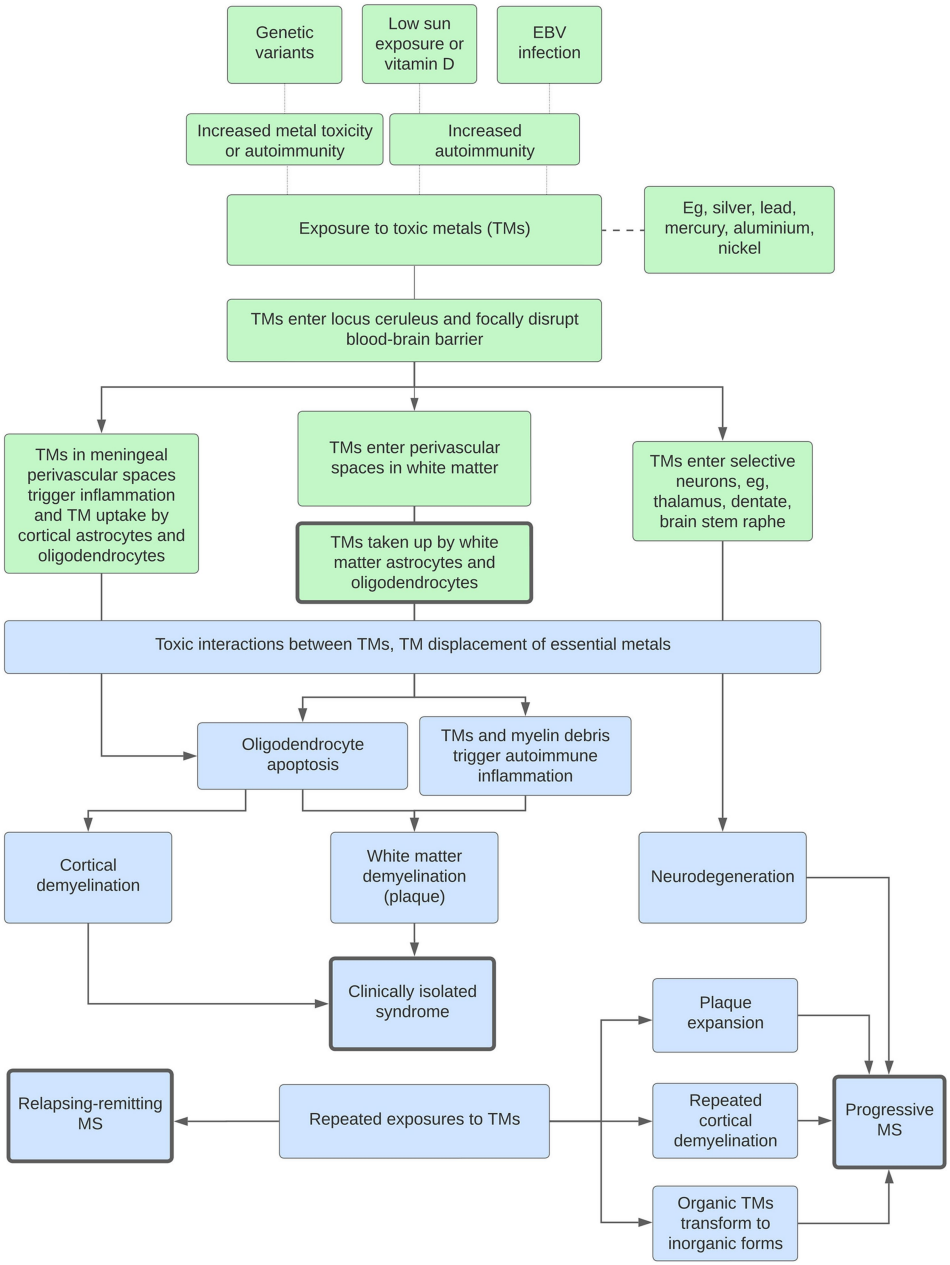
Potential roles played by toxic metals (TMs) in MS. Genetic variants, low sun exposure and/or vitamin D, or Epstein–Barr virus (EBV) infection increase susceptibility to metal toxicity or autoimmunity. *White matter demyelination*: The blood–brain barrier is focally disrupted by a lack of noradrenaline from toxic metal-containing locus ceruleus neurons. This allows toxic metals to enter perivascular spaces in the white matter, followed by uptake into astrocytes and oligodendrocytes, oligodendrocyte apoptosis, and secondary autoimmune inflammation. Interactions between multiple toxic metals, and displacement of essential metals, increase toxicity. *Clinically isolated syndrome*: With no further toxic metal exposure, only one episode of demyelination occurs. *Cortical demyelination*: A similar pathway originating in the perivascular spaces of the meninges and cortex results in cortical demyelination. *Relapsing–remitting MS*: Repeated exposures to toxic metals lead to further episodes of demyelination. *Progressive MS*: Progressive disease is caused by combinations of (i) toxic metal accumulation in neurons causing neurodegeneration, (ii) toxic metals at plaque margins causing expansion, (iii) increased episodes of cortical demyelination, and (iv) the transformation of organic to more toxic inorganic metals.

The toxic metal hypothesis can account for many of the epidemiological and clinicopathological manifestations of MS, as outlined in the flowchart in Figure 8. This proposes how, on a background of increased susceptibility to autoimmunity, toxic metals could play a role in both white matter and cortical demyelination, and in the different clinical forms of MS (isolated, relapsing–remitting, and progressive).

#### 3.3.2. Amyotrophic lateral sclerosis

**Figure 9:**
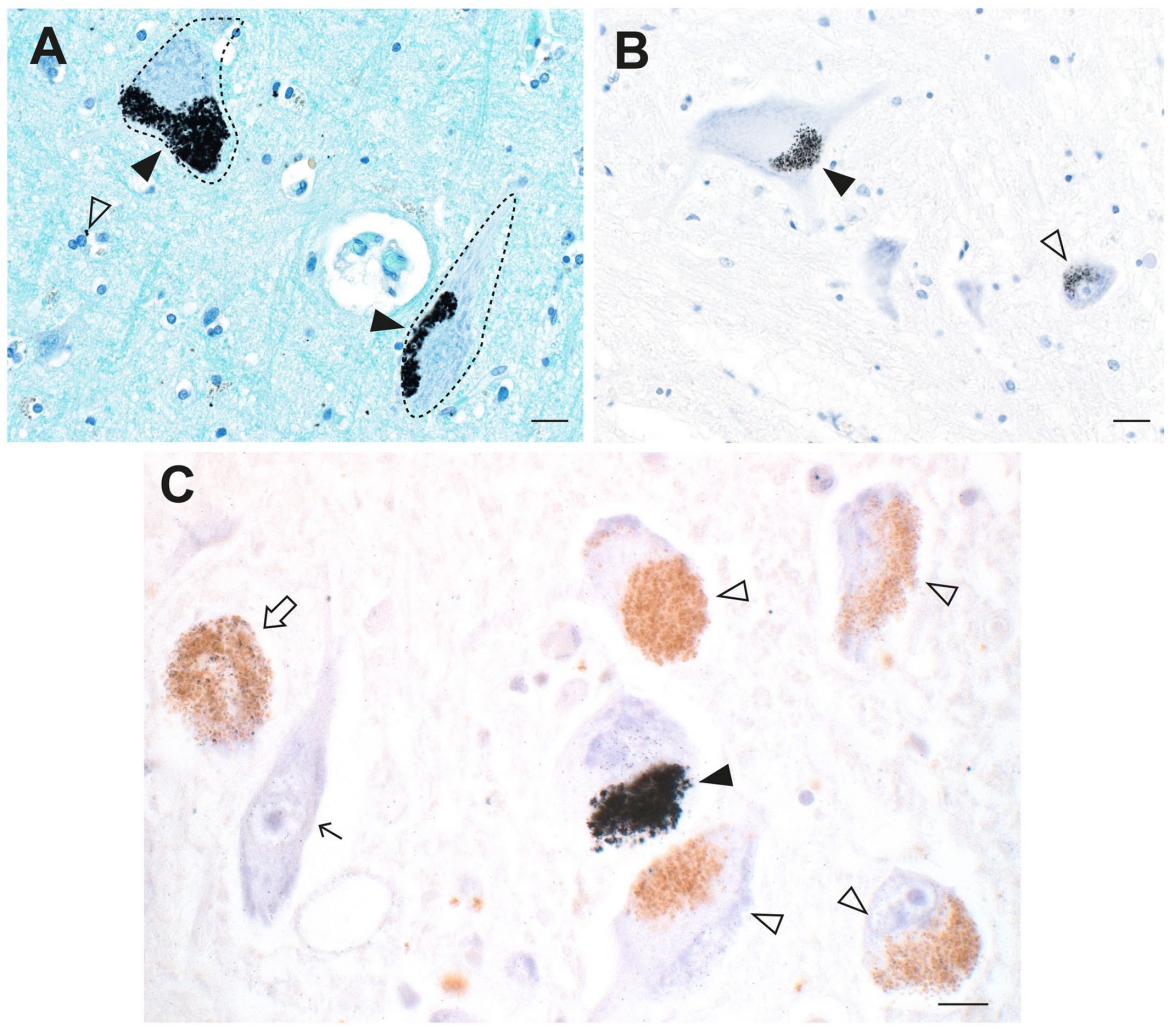
Toxic metals in upper and lower motor neurons and in ALS locus ceruleus neurons. **(A)** Dense AMG™ staining is seen in two corticomotoneurons (closed arrowheads) in the frontal motor cortex from a person with Parkinson disease. Nearby oligodendrocytes contain AMG™ deposits (open arrowhead). **(B)** AMG™ staining is seen in a spinal motor neuron (closed arrowhead), as well as in a small interneuron (open arrowhead) in a control spinal cord. **(C)** In the locus ceruleus of a person with ALS, dense AMG™ staining obscures the neuromelanin in one neuron (closed arrowhead). Sparse AMG™ grains are present in the neuromelanin of another neuron (open arrow). Other neuromelanin-containing neurons (open arrowheads), and a neuron without neuromelanin (thin arrow), are AMG™-free. Methods: **A** adapted from (25), **B** from (158), **C** (18). Bars = 20 μm.

In ALS both corticomotoneurons (upper motor neurons, Betz cells) in the frontal motor strip, and spinal and brain stem motor neurons (lower motor neurons) progressively degenerate (3). Elemental mapping of toxicants in motor neurons from people with ALS is impractical because of the widespread loss of these neurons by the time of autopsy. However, an examination of tissue from non-ALS controls indicates the propensity of motor neurons to take up toxicants. A striking feature found in many control subjects is the specific uptake of AMG™ in corticomotoneurons, together with AMG™ in nearby astrocytes and oligodendrocytes (Figures 2D, 9A). No other neocortical neurons in these samples showed AMG™ uptake. Unfortunately, corticomotoneurons are often not detected in routinely autopsied brains since special techniques are required to reliably locate them (159). Toxic metals such as mercury can cause neuronal hyperexcitability (160), and corticomotoneuron hyperexcitability is postulated as a primary mechanism of motor neuron cell death in ALS (161). Although spinal motor neurons have largely disappeared at time of death in ALS, studies of spinal cords from non-ALS controls have found AMG™ in both alpha spinal motor neurons (65) and in nearby inhibitory interneurons (158) (Figure 9B). Damage to inhibitory interneurons could increase excitotoxic damage to these spinal motor neurons.

**Figure 10:**
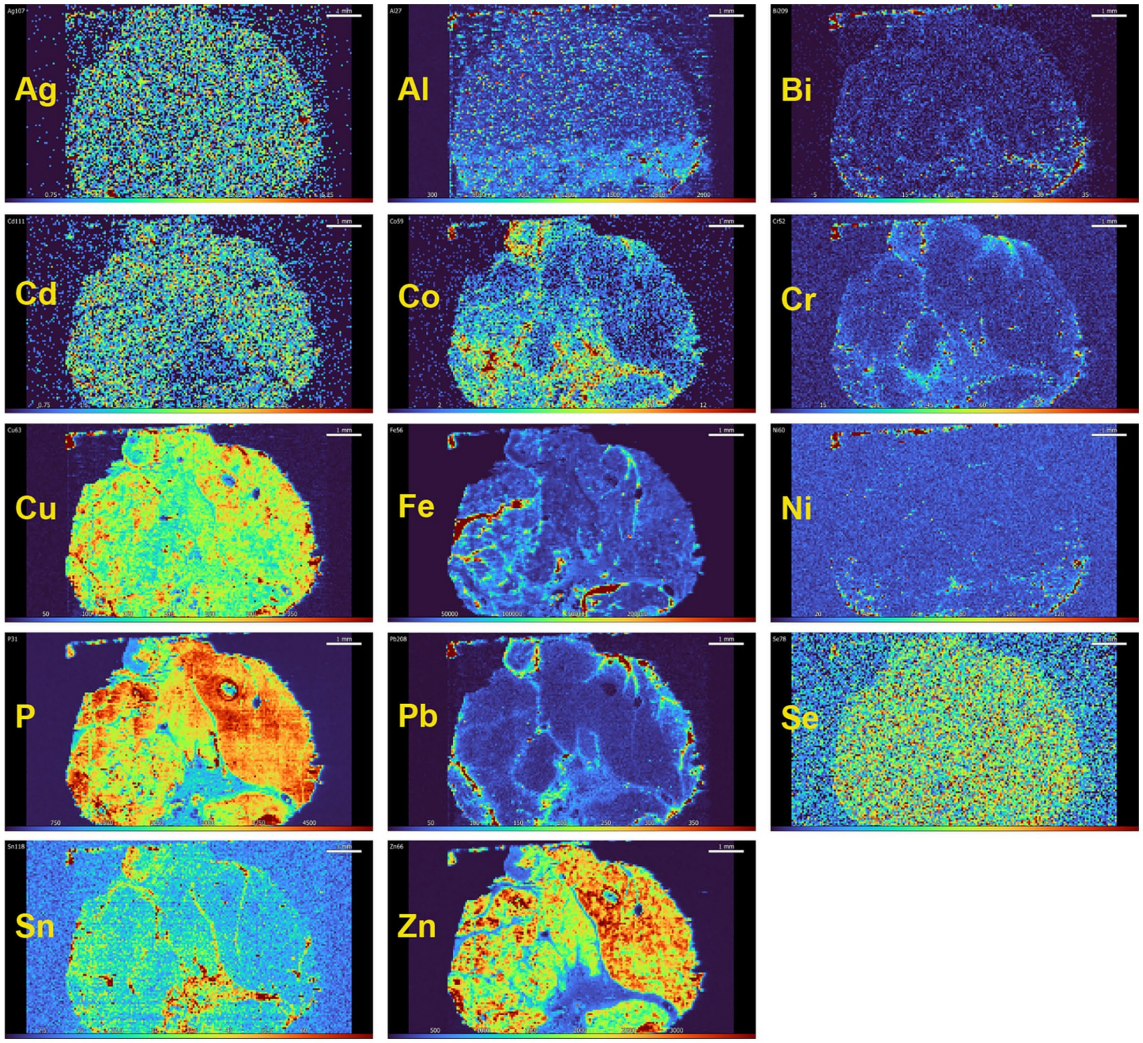
Toxic and essential metals in human striated muscle. In this muscle from a person with no neurological disorder, essential elements, such as copper (Cu), phosphorus (P), selenium (Sn) and zinc (Zn) are present in all myofibres. Some toxic metals, such as silver (Ag) and cadmium (Cd) are also seen in most myofibres. The toxic metals lead (Pb), cobalt (Co), chromium (Cr) and tin (Sn) are present in the fibrous connective tissue between muscle fascicles, which is supplied with large blood vessels. Iron (Fe) is present in blood vessels because of the high iron content of red blood cells. Method: LA-ICP-MSI as used in (26).

The finding of toxic metals in human spinal motor neurons is consistent with animal experiments showing that either intramuscular or systemically administered mercury can be taken up by striated muscles and be retrogradely transported *via* motor axons to spinal and brain stem motor neuron cell bodies, bypassing the blood–brain barrier (10). Even low doses of mercury vapor, below WHO guidelines for toxicity, can be deposited in mouse spinal motor neurons (162). This raises the possibility that toxicants can enter human spinal motor neurons from muscle (163) and contribute to spinal motor neuron loss in ALS (23). High-level exercise appears to be a risk factor for ALS (164), which could be explained by increased transport of toxicants from active muscle to spinal motor neurons. Striated muscle, being a relatively stable tissue, could retain toxic metals for long periods of time. Preliminary studies using LA-ICP-MSI on human muscle suggest this could becase (Figure 10), suggesting that elemental analysis of minimally-invasive needle muscle biopsies might give an indication of past toxicant exposures.

AMG™ staining in locus ceruleus neurons (Figure 9C) is seen significantly more often in ALS patients than in controls (19). The locus ceruleus innervates many cell types that are affected in ALS, so ALS could result from toxicant-induced interactions between the locus ceruleus and motor neurons (18).

The phenotype in ALS differs considerably between patients, with variability in upper and lower motor neuron involvement, cognitive impairment, and extrapyramidal, cerebellar, sensory and autonomic involvement (165). Different distributions of intracellular toxicants could underlie some of this disease variation. In addition, puzzling findings in the 10% of ALS cases that are familial (3) are that the identified germline mutations are present in all cells but affect motor neurons predominantly, the mutations are present at birth but the motor neuron loss manifests only in later adult life, and within families with the same mutation, the age of disease onset can vary markedly. A variable uptake of slowly accumulating metal toxicants within motor neurons could explain many of these features.

**Figure 11:**
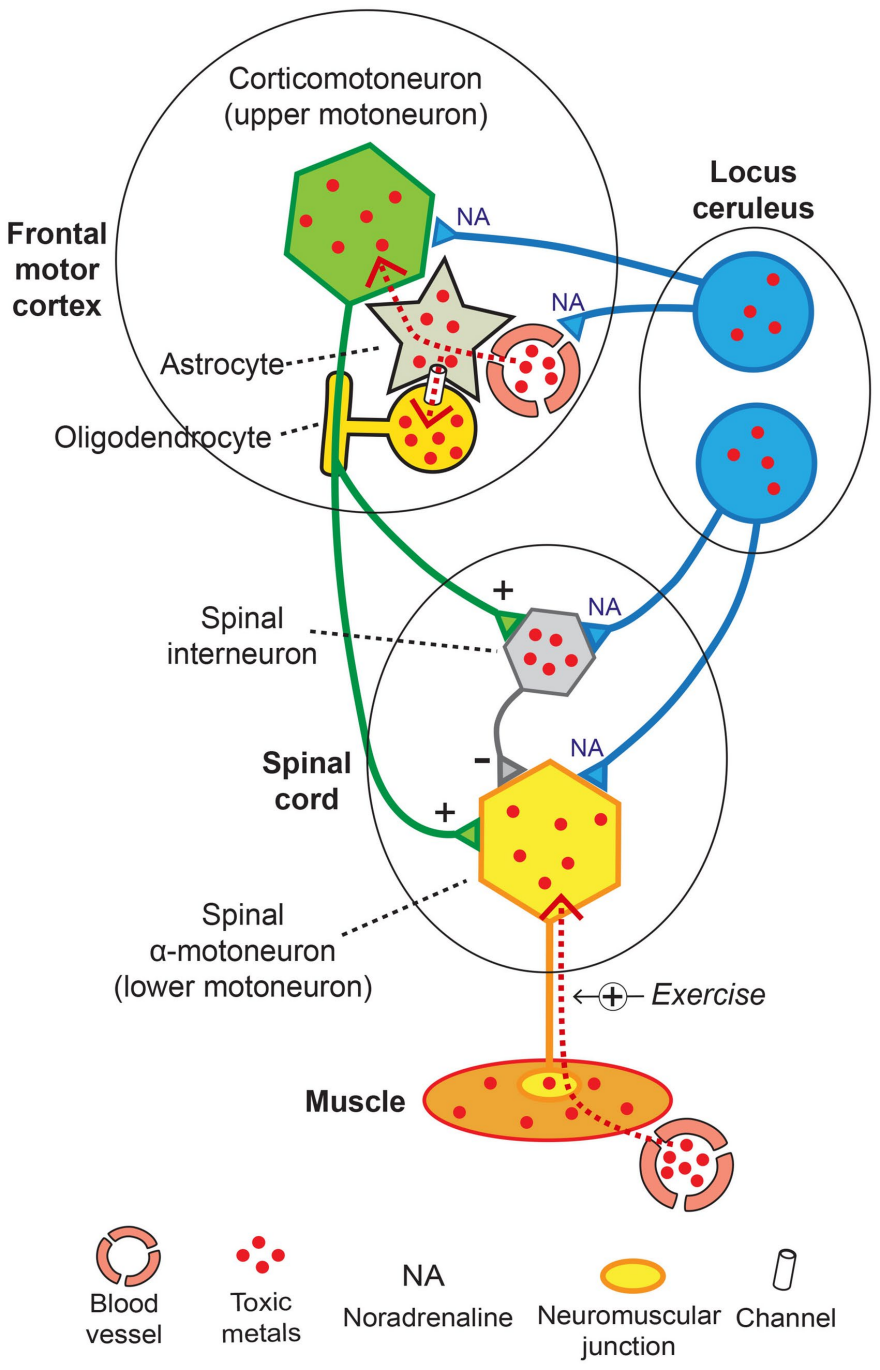
The toxic metal hypothesis in ALS. Damage to locus ceruleus neurons weakens the blood–brain barrier and increases the susceptibility of motor neurons to toxicant damage. Toxic metals enter corticomotoneurons and nearby oligodendrocytes *via* astrocytes. Spinal motor neurons undergo hyperexcitability from toxicant-induced damage to corticomotoneurons and inhibitory interneurons and are loaded with toxicants by retrograde axonal uptake from muscles. Normal transmission: + excitatory, − inhibitory. Exercise +: toxic metal uptake increased with exercise. Diagram adapted from (158).

The toxic metal hypothesis is compatible with most features of ALS (Figure 11). This hypothesis proposes that toxic metals in locus ceruleus neurons set the stage by causing noradrenaline deficits that both weaken the blood–brain barrier and increase the sensitivity of motor neurons to damage. Toxic metals then enter corticomotoneurons *via* astrocytes, and oligodendrocyte uptake of toxic metals demyelinates corticomotoneuron axons. Spinal motor neurons could suffer a quartet of insults, with (i) corticomotoneuron toxicant-induced hyperexcitability, (ii) toxicant-containing spinal inhibitory interneurons causing further excitability, (iii) toxicants transferred from the circulation *via* muscle, damaging lower motor neurons, and (iv) decreased neuroprotection from toxicant-containing locus ceruleus neurons (Figure 11).

#### 3.3.3. Concurrent ALS and MS

**Figure 12:**
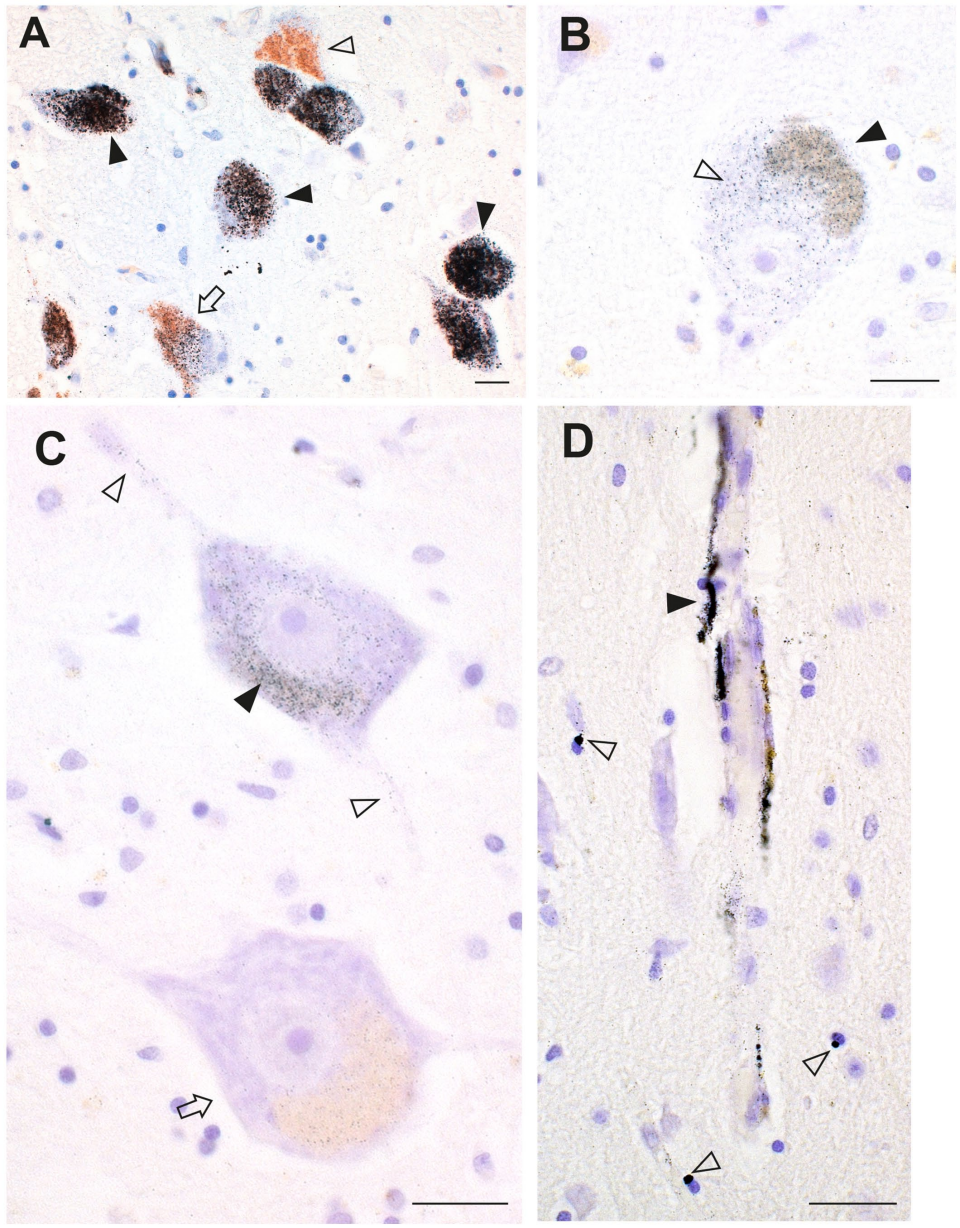
Toxic metals in a patient with concurrent ALS and MS. **(A)** Most locus ceruleus neurons contain dense black AMG™ (closed arrowheads). Some neurons have no (open arrowhead) or only slight (arrow) AMG™ staining. **(B)** A corticomotoneuron cell body has AMG™ in both the lipofuscin (closed arrowhead) and in the remaining perikaryon (open arrowhead). **(C)** One slightly shrunken spinal motor neuron has AMG™ in the perikaryon (closed arrowhead) and in its processes (open arrowheads). An adjacent motor neuron (arrow) contains no AMG™ (arrow). **(D)** Brain microvessels have AMG™ in their walls (closed arrowhead). Nearby oligodendrocytes or pericytes have small paranuclear AMG™ deposits (open arrowheads). Methods: **A–D** (18). Bars = 20 μm.

Pathologically-confirmed concurrences of ALS and MS have occasionally been reported (166). No shared common genetic variants in ALS and MS have been found (167), but the toxic metal hypothesis predicts that toxicants would be present in such cases. A person was found at autopsy to have the pathological features of both ALS and MS. Autometallography of brain samples showed dense AMG™ deposits in locus ceruleus neurons, in numerous brain capillary walls, and in oligodendrocytes and pericytes, as well as in both corticomotoneurons and spinal motor neurons (Figure 12). Toxicants therefore may have contributed to both the ALS and MS pathology in this patient.

#### 3.3.4. Parkinson disease

Neuromelanin-containing neurons in both the locus ceruleus and the substantia nigra have previously been shown to contain toxic metals such as mercury and lead, as well as physiological metals such as iron and copper in toxically-reactive forms (149, 168–171). These pigmented neurons have autolysosomes containing neuromelanin which bind and retain toxic metals such as mercury (that attaches to -SH groups and can react with other metals) for long periods of time (29, 149, 170). Mercury has also been found to cause pathology in neurons in the premotor cortex and cerebellum (170, 172). Noradrenaline from the locus ceruleus appears to protect substantia nigra neurons from damage (68, 173), so toxicant damage to locus ceruleus neurons could both facilitate toxic metal entry into the substantia nigra neurons and augment the toxicity of any toxicants already within these neurons (25).

**Figure 13:**
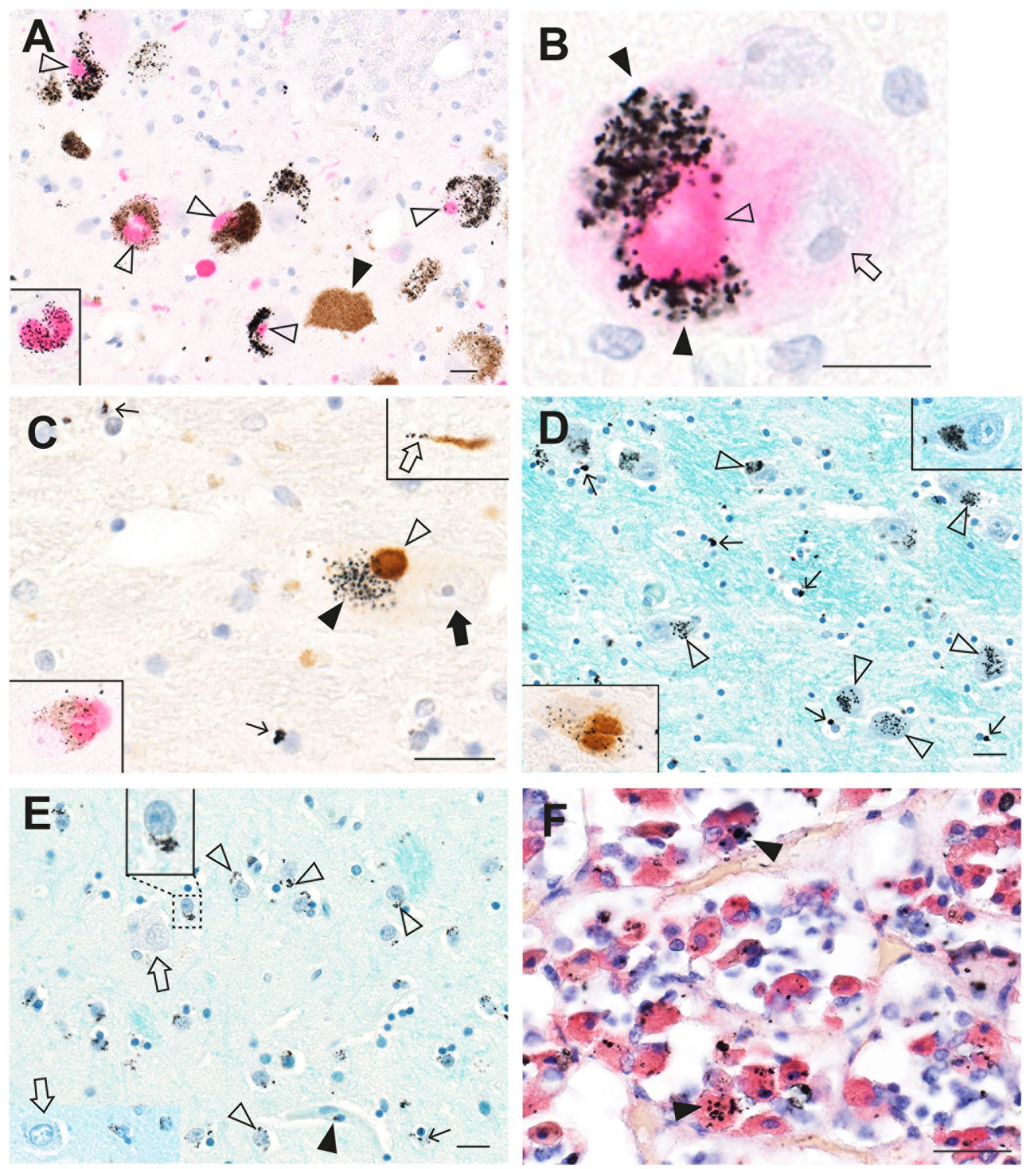
Toxic metals in PD brains and pituitary glands. **(A)** In locus ceruleus neurons, magenta a-synuclein immunostaining shows co-localization of Lewy bodies (open arrowheads) with black-staining AMG™. No Lewy bodies are present in a neuron not containing AMG™ (closed arrowhead). The inset shows AMG™ within a magenta-stained Lewy body. **(B)** A locus ceruleus neuron contains a magenta-stained Lewy body (open arrowhead) partially surrounded by dense AMG™ (closed arrows), adjacent to the nucleus (arrow). **(C)** In the substantia nigra, brown a-synuclein immunostaining shows co-localization of a Lewy body (open arrow) with black AMG™ (closed arrow) in a neuron (nucleus shown by closed arrow). A brown Lewy neurite (upper inset) contains a few black AMG™ grains (open arrow). Scattered oligodendrocytes have small AMG™ deposits (thin arrows). Left inset: AMG™ staining co-localizes with a magenta-stained Lewy body. **(D)** In the thalamus, AMG™ grains are present in the cytoplasm of most neurons (open arrowheads). Magnified view of one neuron shown in upper inset. Numerous oligodendrocytes have small AMG™ deposits (arrows). AMG™ staining co-localizes with a brown Lewy body in one neuron (lower inset). **(E)** In the putamen, AMG™ grains (open arrowheads) are present in medium-sized neurons (one enlarged in the upper inset). Large neurons (open arrows) contain no AMG™. Small AMG™ deposits are present in scattered oligodendrocytes (arrow) and pericytes (closed arrowhead). **(F)** In the anterior pituitary, AMG™ grains are present in scattered, red-immunostained growth hormone-containing cells (arrowheads). Methods: **A–E** adapted from (25), **F** (55). Bars = 20 μm.

In the brains of people who had PD, AMG™ staining has been seen in many neurons affected by the disease, such as surviving neurons in the substantia nigra and locus ceruleus, and those in the frontal motor cortex, striatum, thalamus, and cerebellum, as well as in oligodendrocytes in white and grey matter (Figure 13) (25). AMG™ co-localized with Lewy bodies and neurites, and AMG™ was prominent in extra-CNS cells, such as chromaffin cells of the adrenal medulla, kidney proximal tubules and thin Henle loops, thyroid follicular cells, and anterior pituitary growth-hormone containing cells.

**Figure 14:**
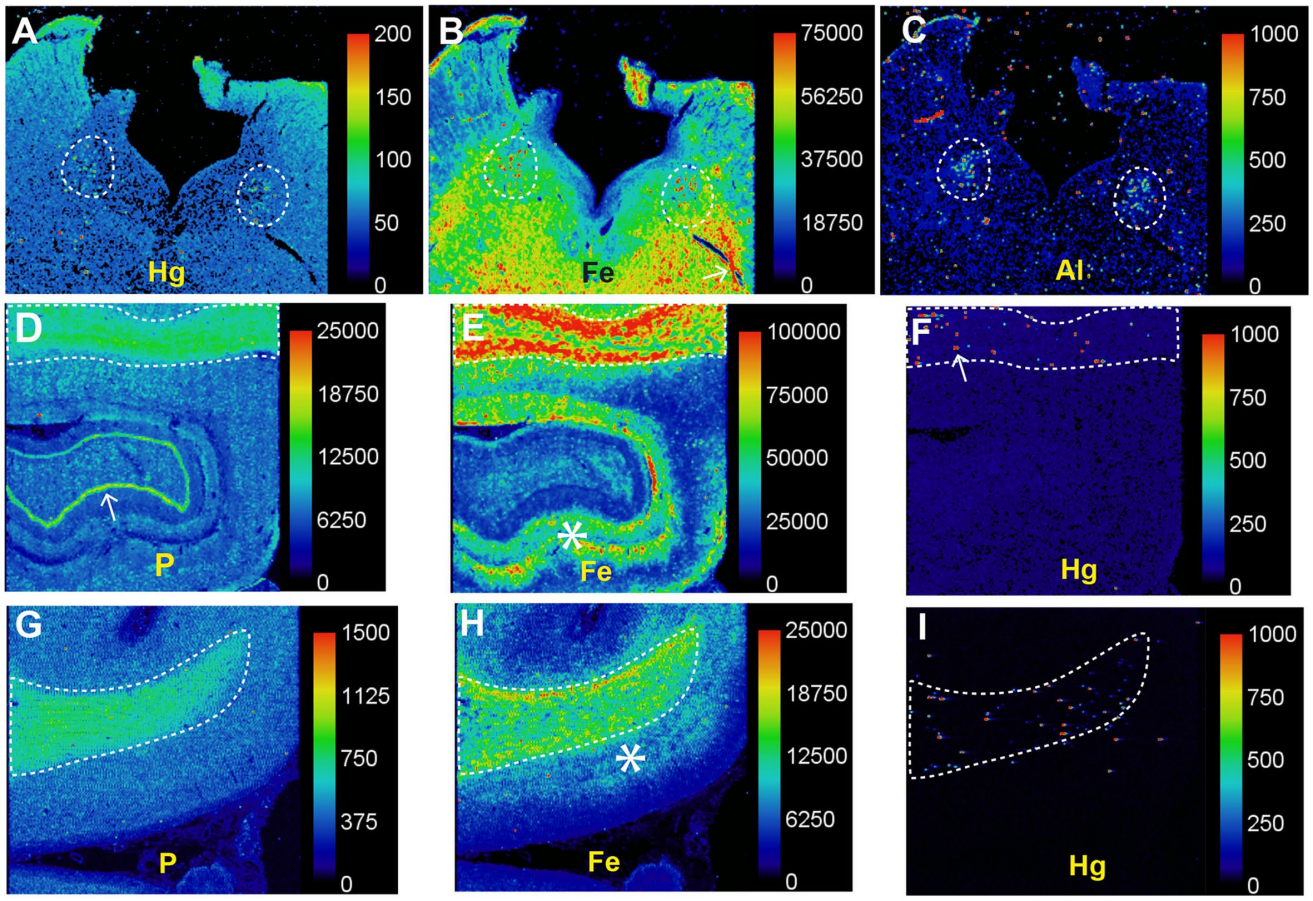
Toxic metals in PD brains detected on LA-ICP-MSI. **(A–C)** Posterior pons. Mercury **(A)**, iron **(B)**, and aluminium **(C)** are prominent in the locus ceruleus (dashed outlines). **(D–F)** Hippocampus. A high nuclear density, shown by the phosphorus image **(D)**, is present in the hippocampal white matter (top, dashed outline) as well as in the dentate gyrus (arrow). A large amount of iron **(E)** is present in the hippocampal white matter (top, dashed outline), and in grey matter adjacent to the dentate gyrus (asterisk). Particulate mercury **(F)** is seen in the hippocampal white matter (arrow) in the dashed outline. **(G–I)** Frontal white matter. The high nuclear density of the frontal white matter (dashed outline) is shown in the phosphorus image **(G)**. The the frontal white matter, shown by the phosphorus image **(G)**, contains more iron **(H)** than the frontal cortex, where most iron is in the deeper cortical layers (*) adjacent to the white matter. Particulate mercury **(I)** is present in the frontal white matter. Scale = counts per second (proportional to abundance). Fig adapted from (25).

In patients with PD LA-ICP-MSI has demonstrated mercury and other potentially toxic metals in the locus ceruleus and high iron levels in the white matter (Figure 14). The co-occurrence of iron and mercury in locus ceruleus neurons, and in the white matter (probably in oligodendrocytes), raises the possibility that interactions between these two metals could damage cells in PD, and could be a reason why iron appears to play a part in many neurodegenerative diseases (28).

A flow diagram modeling how toxic metals could be responsible for many of the clinicopathological features of PD has been published (25). In this model, mercury was used as an example of how exposure to a toxic metal, on a background of genetic susceptibilities, could result in the varied manifestations of PD.

#### 3.3.5. Alzheimer disease

It is difficult to look for cellular evidence for the toxic metal hypothesis in AD autopsies because of the widespread loss of CNS cells that takes place in the years between AD onset and death. Any toxic metals found in severely affected AD brains could be those taken up secondarily by damaged cells (mineralization), rather than representing a primary toxicant-based injury. There is, however, indirect evidence that toxic metals could play a role in AD. (i) Toxic metals can be found in locus ceruleus neurons from the second decade of life onwards (43) and the locus ceruleus is the region of the brain first affected by AD, with tangle pathology detected in these neurons from before puberty or in early young adulthood (174). (ii) Cerebral neurofibrillary pathology in AD is first located in cells in superficial layers of the medial temporal lobe (175). This is close to the region where intracellular toxic metals are present in the superficial cortex adjacent to leptomeningeal venules (Figure 4E). (iii) Dementia is common in the later stages of PD, and while most of this appears to be due to Lewy body pathology, amyloid plaque pathology is found in up to one-third of patients with PD (176). This suggests pathological commonality between PD and AD and accords with other aspects of AD shared by neurodegenerative disorders that could be toxicant-induced (Table 1). (iv) One clue to toxicant-induced AD could be the finding that mercury and iron co-localize in locus ceruleus neurons and in hippocampal white matter oligodendrocytes in PD brains (Figure 14), given the early involvement of the hippocampus in AD, the suspected role of iron in a variety of neurodegenerative disorders (28), and the realization that synergistic interactions between intracellular toxic metals are common (12).

## 4. Testing the toxic metal hypothesis

Finding evidence to support the toxic metal hypothesis could take several avenues. (i) Statistically robust human cellular elemental mapping studies require large autopsy cohorts of people both with and without neurological disorders. This will be challenging, given the universal decline in autopsy numbers in recent years. (ii) Advanced equipment for LA-ICP-MSI is now available which will enable faster analysis of multiple elements in large numbers of samples. This will help assess the contribution of combinations of toxic metals to neurological disorders. (iii) Efforts are needed to develop new imaging techniques that can locate toxic metals in the brain in life, to enable anti-toxicant therapy early in the course of the disease. (iv) Assessment of a patient’s toxic metal burden during life could be made by elemental mapping of non-CNS tissue such as striated muscle that is likely to retain toxicants for long periods of time and that can be safely biopsied (Figure 10). This could enable early treatment for toxicant overload, though chelation therapy for toxic metals has possible side-effects of tissue re-distribution that need to be considered (177). (v) Finding genetic susceptibilities to metal toxicants, such as by using Drosophila models (178), could uncover predispositions to toxicant-induced neurological diseases and could aid in preventative measures by avoiding toxicant exposures.

## 5. Conclusion

In conclusion, combinations of environmental toxic metals are found commonly in the brains of people with neurological disorders, and in non-neurological controls. Toxic metals can be detected in human astrocytes, oligodendrocytes, and neurons, as well as in a variety of other cells implicated in neurological disorders. The toxic metal hypothesis can explain the multitude of clinicopathological features present in people with common neurological conditions. Underlying genetic variants, and different combinations of toxic metals, could be the reason exposure to toxic metals results in a variety of neurological disorders. While further work to test the toxic metal hypothesis for neurological diseases is undertaken, to protect our nervous systems from disease it would be prudent to make intensive efforts to greatly limit industrial, mining, manufacturing activities and fossil fuel burning that release toxic metals into the air, water, and soil (179).

## Data availability statement

The original contributions presented in the study are included in the article, further inquiries can be directed to the corresponding author.

## Ethics statement

The studies involving human participants were reviewed and approved by Human Research Committee, Sydney Local Health District (Royal Prince Alfred Hospital Zone). Written informed consent for participation was not required for this study in accordance with the national legislation and the institutional requirements.

## Author contributions

RP originated the hypothesis, supervised the autometallography and immunohistochemistry, undertook the photomicroscopy, created the flowchart, drew the figure diagrams, and drafted the manuscript. DB performed the LA-ICP-MSI and analyzed the results. Both authors revised and approved the submitted article.

## Funding

RP is supported by the Aimee Stacey Memorial and Ignacy Burnett Bequests.

## Conflict of interest

The authors declare that the research was conducted in the absence of any commercial or financial relationships that could be construed as a potential conflict of interest.

## Publisher’s note

All claims expressed in this article are solely those of the authors and do not necessarily represent those of their affiliated organizations, or those of the publisher, the editors and the reviewers. Any product that may be evaluated in this article, or claim that may be made by its manufacturer, is not guaranteed or endorsed by the publisher.
